# Facial beauty affects implicit and explicit learning of men and women differently

**DOI:** 10.3389/fpsyg.2015.01124

**Published:** 2015-08-05

**Authors:** Eleni Ziori, Zoltán Dienes

**Affiliations:** ^1^Department of Psychology, Faculty of Philosophy, Education and Psychology, School of Philosophy, University of IoanninaIoannina, Greece; ^2^Sackler Centre for Consciousness Science and School of Psychology, University of SussexBrighton, UK

**Keywords:** AGL, implicit learning, explicit learning, beauty, motivation, incentive salience

## Abstract

The present work explores the unconscious and/or conscious nature of learning attractive faces of same and opposite sex, that is, of stimuli that experimental and neuroimaging research has shown to be rewarding and thus highly motivating. To this end, we examined performance of men and women while classifying strings of average and attractive faces for grammaticality in the experimental task of artificial grammar learning (AGL), which reflects both conscious and unconscious processes. Subjective measures were used to assess participants’ conscious and unconscious knowledge. It was found that female attractiveness impaired performance in male participants. In particular, male participants demonstrated the lowest accuracy while classifying beautiful faces of women. Conversely, female attractiveness facilitated performance in female participants. The pattern was similar for conscious and unconscious knowledge. Presumably, objects with high incentive salience, as are beautiful faces, captured resources, which were used in task relevant versus task irrelevant ways by women versus men. The present findings shed light on the relation of conscious and unconscious processing with affective and reward-related stimuli, as well as on gender differences underlying this relation.

## Introduction

The motivational value of facial beauty has been evidenced both by laboratory-based research (e.g., Levy et al., 2008) and by numerous neuroimaging studies (e.g., Aharon et al., 2001; Kampe et al., 2001; O’Doherty et al., 2003; Kranz and Ishai, 2006; Ishai, 2007). More specifically, functional brain imaging studies have shown that facial beauty evokes activation in brain regions that are involved in stimulus-reward associations (e.g., in the orbitofrontal cortex or the ventral striatum) and thus form key structures that support affect and emotion. Thus, beautiful faces can be thought of as rewarding stimuli that activate brain regions involved in the processing of other primary rewards (e.g., food and sexual contact; see e.g., Arana et al., 2003) or secondary rewards (e.g., monetary gain; see e.g., Kringelbach and Rolls, 2004). Further, another main finding of the above studies (O’Doherty et al., 2003; Kranz and Ishai, 2006; Ishai, 2007) is that attractive female faces elicit stronger motivational/reward regions activation than attractive male faces ones in heterosexual men, and that attractive male faces evoke stronger activation than attractive female faces in heterosexual women. This pattern of neural activation reflects the emotional valence assigned to attractiveness, which plays a significant role in mating success.

In a similar vein, Levy et al.’s (2008) study, which used laboratory performance tasks, showed that facial beauty attracts attention and has high incentive value. Levy et al. (2008) examined the effect of gender on the processing of facial beauty by asking male and female participants to control the viewing time of average or beautiful faces of men and women, as well as to rate their attractiveness. The two groups of participants gave similar ratings of heterosexual facial attractiveness. However, men had the longest viewing time for beautiful female faces, which was much longer than the corresponding time that women had for beautiful males. Further, women had an increased viewing time for both beautiful male and female faces, whereas men focused only on beautiful female faces. The authors interpret their findings in terms of gender-specific incentive sensitization mechanisms.

There is substantial evidence that human behavior is to a large extent driven by motives/rewards and goals (e.g., McClelland, 1985; Custers and Aarts, 2005; Aarts et al., 2007). A key issue is whether motive driven behavior is a conscious process or whether motives can be activated unconsciously and unintentionally. Traditionally, motives were considered to involve an effortful and intentional process, which is activated with conscious awareness. Further, implicit learning was traditionally equated with a passive, unselective learning process that occurs without directing people’s attention to the task at hand and regardless of the relevance of stimulus structure to people’s goals or specific motivations (e.g., Hayes and Broadbent, 1988; Lewicki et al., 1992). However, contemporary empirical studies have shown that implicit learning is sensitive to what is perceptually attended (e.g., Tanaka et al., 2008; Eitam et al., 2009; Eitam and Higgins, 2010; Kiyokawa et al., 2012; Norman and Price, 2012) and so may well be sensitive to the incentive salience of different stimuli.

Implicit learning is the acquisition of unconscious knowledge (e.g., Reber, 1967, 1989; cf. Stadler and Frensch, 1994; Frensch, 1998). In the present work, we are focusing on one of the most widely known experimental tasks that have been used to study implicit learning, namely artificial grammar learning (AGL).

In a typical AGL task (e.g., Reber, 1967), participants have to simply observe a set of symbol strings, which are constructed on the basis of a complex rule system (i.e., a finite state grammar). After this training phase, participants are informed for the first time that the strings they had just observed were based on certain complex rules, without receiving any further information regarding the nature of these rules. Next, they are asked to classify new strings, only half of which obey the rules of the first phase and are thus called grammatical (G) in opposition to the other half, which are called non-grammatical (NG) as they do not obey the rules. A typical finding of studies applying the AGL task is that participants distinguish between G and NG strings successfully without being able to report the rules they relied on (e.g., Reber, 1967, 1976). The AGL paradigm constitutes a type of implicit learning in that learning in the context of this experimental paradigm occurs without intention and results in knowledge that is not directly available to conscious introspection.

The AGL task has traditionally used strings of letters, shapes and other symbols involving no meaningful pre-existing associations. More recent variations employed strings of stimuli eliciting people’s prior knowledge and expectations, and showed that implicit AGL reflects selective processes and may be affected by people’s prior (geography) knowledge (Ziori et al., 2014), goals (Eitam et al., 2009), and motivational relevance (Eitam and Higgins, 2010, 2014; Eitam et al., 2013; cf Eitam et al., 2014 for evidence of implicit learning of faces when they were task irrelevant). The present work aims to extend the above AGL research in a different knowledge-rich context and examine whether the processing of beautiful faces, which have been shown to trigger the reward center of brain, is associated more with implicit or with explicit knowledge.

It should be noted at this point that many of the above studies that have provided evidence of the reward value of facial beauty draw on Berridge ’s (1996, 2000) distinction between “liking,” a positive affect that corresponds to assessments of attractiveness and “wanting” or incentive salience, namely the psychological component of reward that corresponds to key pressing measures. “Liking” corresponds to the process of hedonic pleasure, that is, to the emotional experience a particular object evokes. “Wanting” as used by Berridge and Winkielman (2003) and Berridge (2009) refers to “incentive salience,” which corresponds to a motivation process that produces seeking behavior and is closely dependent on the presence of the reward itself or of a cue that functions as a reminder of that reward. “Wanting” and “liking” are two constructs that are closely related in that when we like something, in most cases, we want it. However, it has been suggested that the two concepts are distinct and neurobiologically dissociable (e.g., Berridge and Robinson, 1998, 2003; Smith and Berridge, 2007).

In the AGL paradigm, motivational and esthetic aspects of beauty are difficult to disentangle. Presumably, both aspects are involved as participants engage both in passive viewing, which is associated more with liking, and in directing their attention to faces that they desire or that work as reward cues, which may be associated with a wanting process.

Many psychologists assume that affective states (hedonic experiences) are in essence conscious. However, according to Wyvell and Berridge (2000), Berridge (2003), and Berridge and Winkielman (2003), wanting and liking are two distinct components of the “reward architecture,” which may operate both explicitly (consciously) and implicitly (unconsciously). While Berridge and Winkielman (2003) acknowledge that wanting as used by most people refers to conscious cognitive desire, their ‘incentive salience “wanting,”’ is a more percept-driven process that does not always need to be accompanied by conscious awareness. Further, they provide evidence of subliminally induced positive affective reactions.

In the present work, we attempt to enlighten the relationship of unconscious processing with affective and reward-related reactions by examining the implicit and/or explicit nature of learning rewarding stimuli (i.e., attractive faces) in the unintentional learning context of the AGL paradigm. To this end, we replaced the original strings of letters used in Dienes and Scott (2005, Experiment 2) with strings of people’s full-face photos. As in the standard AGL task, participants first observed the strings of faces, and in the test phase that followed, they judged them for grammaticality.

As mentioned above, stimuli with high incentive salience are generally thought to capture a greater amount of attention resources and generate greater behavioral effort in comparison to stimuli of lower reward value (e.g., Levy et al., 2008; see also Langlois et al., 1987, 1991). Studies on the role of attention in implicit learning, in general, or in AGL, in particular, have resulted in divergent findings and conclusions. This blurred picture may be partly due to the different meanings attributed to the term attention (e.g., one that equates attention with cognitive effort and the availability of resources vs. one that views attention as a selective process; see Jiang and Chun, 2003). For instance, it has been suggested that executive resources are not required for implicit AGL (Dienes and Scott, 2005; or are even detrimental to implicit learning, Janacsek and Nemeth, 2013; Nemeth et al., 2013), but perceptual resources are required (e.g., Tanaka et al., 2008; Eitam et al., 2009; Kiyokawa et al., 2012).

So, stimuli with high incentive salience, as are attractive faces of opposite or even of same sex (see the General Discussion for possible hypotheses on this issue), are expected to guide participants’ attention, which, in turn, could affect performance, and thus the acquisition of implicit and explicit knowledge in different ways. If incentive salience increases perceptual processing, implicit learning should increase for attractive rather than average faces, especially of the opposite sex. Conversely, increased incentive salience might have the opposite effect on performance. For instance, according to Easterbrook’s (1959) influential hypothesis, arousal, or increased drive leads to attentional narrowing, namely to a reduction in the “range of cue utilization” or “breadth of perceptive field” (see also Gable and Harmon-Jones, 2008, 2010a,b; Harmon-Jones and Gable, 2009). Such an attentional narrowing might interfere with perceptual processing, and accordingly impair implicit learning for attractive rather than average faces, especially of the opposite sex. The present work aims at clarifying the two opposing possibilities concerning the effect of incentive salience of beauty on perceptual resources and consequently on implicit AGL.

Finally, if incentive salience increases only executive processing, implicit learning should be unaffected (or even impaired: Nemeth et al., 2013), and the above possibilities might hold only for explicit learning; thus attractive faces, especially of the opposite sex, should enhance explicit learning if they allocate attentional resources in a global manner or reduce it if they act in a more local way focusing, for instance, on the details of the faces.

## Materials and Methods

### Design and Participants

Three main between-participant independent variables were used, namely participants’ gender (female vs. male), beauty (average vs. beautiful), and gender (women vs. men) of the faces of people depicted in the photos. The present study used the two grammars applied in Dienes and Scott (2005, Experiment 2). Half of the participants were trained on grammar A and the other half on grammar B. The test phase consisted of an equal number of grammar A and grammar B items, which were the same for all participants. The former test items were grammatical and the latter ungrammatical for participants trained on grammar A, while the reverse held for participants trained on grammar B. Thus, grammar type was a fourth between-participant independent variable.

One-hundred and twenty-six undergraduate students from the University of Ioannina (64 females and 62 males) participated voluntarily. One fourth of the participants in each of the two gender categories were allocated in each of the four gender of faces by beauty cells. In each of the above four cells, half of the female participants (i.e., eight participants) and almost half of the male participants (i.e., either seven or eight) were trained on grammar A and the other half on grammar B. A criterion that had to be met for participation in the present study was heterosexuality (or at least a clear attraction to the opposite sex). Eight participants (two females and six males) were excluded from the analyses, as the responses they provided on two questions asking about their physical attraction and their sexual behavior toward people of the opposite sex suggested that the above criterion was not clearly satisfied. In particular, at the end of the experiment, all participants had to answer the following two questions: (A) To whom are you physically attracted? (B) Which gender you had (or would like to have) a sexual relationship with? The answers to both questions were: (1) other sex only, (2) other sex mostly, (3) other sex somewhat more, (4) both sexes equally, (5) same sex somewhat more, (6) same sex mostly, (7) same sex only. The eight participants were excluded because they had chosen replies four and upward in the two questions. So, the data of a total of 118 students (62 females and 56 males) were included in the analyses of the present experiment.

The present research was ethically approved by the University of Ioannina Research Committee. Informed consent was obtained from all participants. At the end of the experimental task, participants were also asked whether they wanted to answer the anonymous questions regarding their sexual preferences and they all accepted. It was emphasized that their replies and personal data would be (and were indeed) treated confidentially.

### Materials

Stimulus construction was based on the two grammars, grammar A and grammar B, used in Dienes and Scott (2005, Experiment 2). The original grammatical strings of each grammar were made up from the letters M, T, V, R, and X, and had a length between five and nine letters. A set of fifteen grammatical strings was repeated three times in different random orders to create the training set of each grammar. Each string length appeared equally often in each training set. The unique test set was formed from a random combination of thirty new strings from each grammar. The proportion of strings of each length was kept constant in the training and test sets.

To create the present strings, we replaced the letters of the original strings with people’s full-face photos, such that each letter corresponded to a unique face in each of the four gender (women vs. men) by beauty (average vs. beautiful) categories of the faces. Thus, there were five faces of each sort (20 faces in total). Each of four groups of men saw a different set of faces (i.e., one saw a set of beautiful male faces, a second one a set of beautiful females, another one saw a set of average males and a last one saw a set of average females). Four groups of women saw exactly the same sets as the above, with each group seeing only one set. The average faces were selected from a collection of images available for psychology experiments (Psychological Image Collection at Sterling, PICS; http://pics.psych.stir.ac.uk). The beautiful faces were selected from international print and digital media excluding faces of famous people.

All the processed photos that were used in the AGL task were pre-tested by 12 participants (six males and six females) that did not take part in the main experiment. In this stimulus test, participants were asked to rate a sample of 60 male and 60 female faces on a scale from 1 = ugly to 7 = beautiful. The faces that were allocated to the categories of the average and the beautiful faces were those that received ratings 3–5 and 6–7 from all participants, respectively. Participants confirmed that none of the selected faces were known or familiar to them.

Microsoft PowerPoint was used to present both training and test strings of faces. All face images were cropped around the hairline and were adjusted to have approximately equal size. Further, all faces had neutral expression with respect to emotion and forward eye-gaze. Each face image was converted to black and white and was placed in a 235 × 314 pixel black background.

### Procedure

In the training phase, all participants were informed that they were about to see sequences of faces of people ordering one drink after the other at a bar, and were asked to observe them carefully. Participants sat at approximately 57 cm from the computer screen. The faces of each training string appeared sequentially in the vertical middle of a black screen, with each face flashing for 0.75 s. When all faces of a string had appeared, the whole string remained on the screen for an extra 1.75 s. The training strings of faces were individually presented on a computer screen and were separated from each other with a blank screen that appeared for 0.75 s.

After training, participants were informed that the strings they had seen followed a complex set of rules, but no specific information was provided regarding the nature of the rules. Then, they were asked to determine which of a new set of face strings followed the rules of the first phase and which did not. After each response, participants had to determine the source of their knowledge by choosing one of the following categories: guess, intuition, familiarity, rules, memory. The following clarifications were given for each category: Choose “guess,” when your response was based on no information whatsoever, that is, when your response was completely random, as if you flipped a coin. Choose “intuition” if you have some confidence in your response, but you have no idea why your response is correct. Choose “familiarity,” if a string seems familiar to you, but you cannot explain why this is so. Choose “rules,” if you feel your response was based on a rule or set of rules that you learned from the first part and that you could report if required. Choose “memory” if it seems to you that your response was based on memory, that is, if you can remember a specific string or part of a string from the first part.

Test strings were presented in the same order for all participants. In addition, test strings appeared in exactly the same manner as the training strings, with the difference that each test string remained on the screen until participants provided their response and knowledge attribution. No corrective feedback was provided.

### Pre-Test Data

The facial attractiveness ratings that the 12 participants of the pre-test gave for the 20 faces used in the present study are presented in **Table 1**. Further, with respect to the attractiveness assessment of the 60 male and the 60 female faces of the initial sample of faces, men’s ratings of male and female faces were (M = 3.4, SD = 0.3) and (M = 3.6, SD = 0.4) respectively, and women’s ratings of male and female faces were (M = 3.4, SD = 0.3) and (M = 3.7, SD = 0.8) respectively.

**Table 1:** Average attractiveness ratings for faces used in the present study.

Gender of participants	Gender of faces	Beauty	M	*N*	SD
Female	Women	Average Beautiful	3.5 6.2	6 6	0.5 0.2
	Men	Average Beautiful	3.8 6.3	6 6	0.8 0.2
Male	Women	Average Beautiful	3.3 6.1	6 6	0.5 0.2
	Men	Average Beautiful	3.7 .3	6 6	0.5 0.1

## Results

Classification accuracy in terms of grammaticality is used to assess learning under the different conditions. Performance on the subjective reports is used to assess the implicit (unconscious) or explicit (conscious) status of the acquired knowledge. For simplicity, in the following analyses, we will report effects testing our predictions that performance will be higher (in terms of the first prediction) or lower (in terms of the second prediction) for beautiful than for average faces and for beautiful faces of the opposite than of the same sex. Effects are tested using Bayes factors, *B*, to assess strength of evidence; *p*-values are also reported so readers can in addition assess significance. A *B* of 3 or above indicates substantial evidence for the alternative rather than the null hypothesis and of 1/3 or below substantial evidence for the null rather than alternative hypothesis. Thus, a *B* between 3 and 1/3 indicates data insensitivity for distinguishing the alternative and null hypotheses (see Dienes, 2008, 2014). *B*s testing group differences are reported for differences justified by our predictions regarding the central issue of this paper, i.e., the effect of same and opposite sex attractiveness on learning. *B*_H(0,6)_ refers to a Bayes factor used to test the hypothesis that there is learning or a difference between groups, represented as a half-normal with a SD of 6% above baseline, against H_0_, the hypothesis of no difference. Following Dienes (2014) when a roughly expected effect size can be specified, it is used as the SD of a half-normal. As the overall mean accuracy for the present data is 56 and 6% was taken as the rough difference expected for specific group comparisons.

For comparisons between two groups, H_1_ *B*_H(0,6)_ refers to Bayes factor testing the hypotheses that performance will be higher for beautiful than for average faces and for beautiful faces of the opposite than of the same sex, whereas H_2_ *B*_H(0,6)_ corresponds to Bayes factor testing the hypotheses that performance will be lower for beautiful than for average faces and for beautiful faces of the opposite than of the same sex.

For three-way interactions, H_1_ suggests that male participants should demonstrate greater learning for beautiful rather than average faces of women rather than men as compared to female participants. H_2_ represents the opposite direction with female participants demonstrating greater learning for beautiful rather than average faces of women rather than men in comparison to male participants. Similarly, for the two-way interactions, H_1_ tests whether learning is greater for beautiful rather than average faces of different than of same gender, whereas H_2_ tests whether learning is greater for beautiful rather than average faces of same than of different gender.

### Classification Performance

**FIGURE 1:**
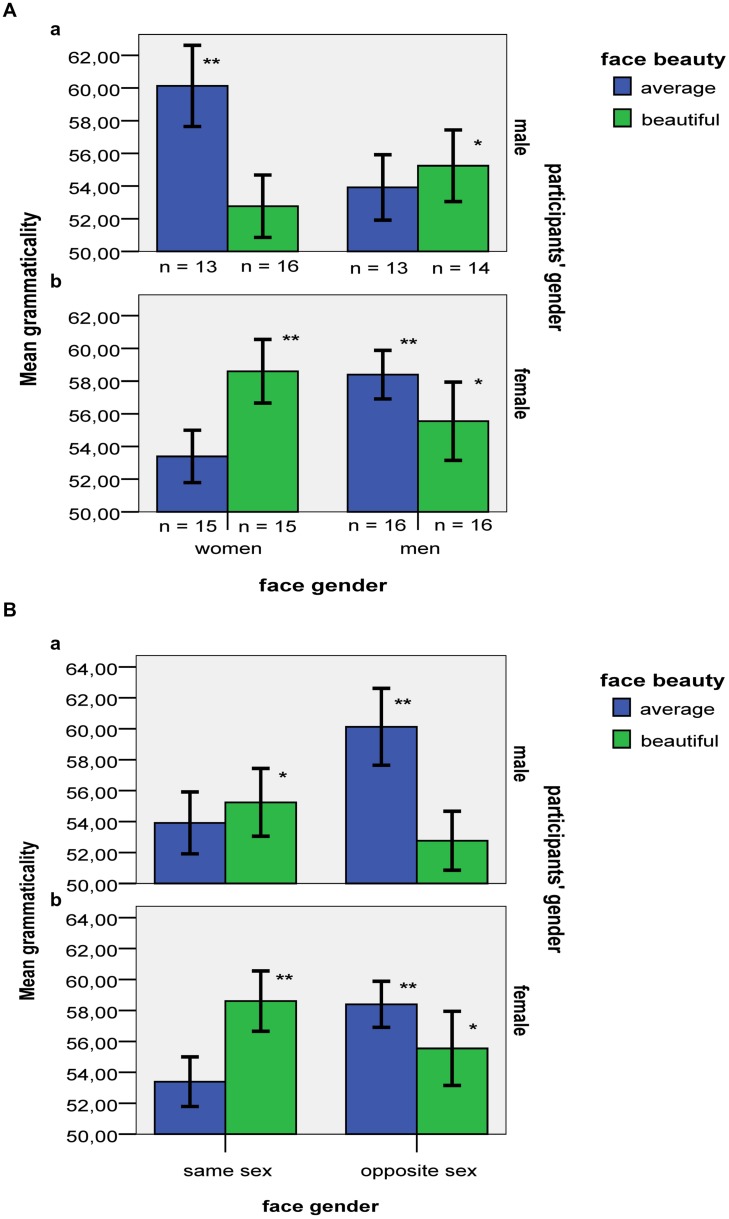
**(A)** Mean grammaticality accuracy of males (a) and females (b) for beautiful vs. average faces of men and women. Error bars indicate ±1 SE. Asterisks denote significant differences from a chance level of 50% (^∗∗^*p* < 0.01; ^∗^*p* < 0.05). **(B)** Mean grammaticality accuracy of males (a) and females (b) for beautiful vs. average faces coded as same vs. opposite to participants’ gender. Error bars indicate ±1 SE. Asterisks denote significant differences from a chance level of 50% (^∗∗^*p* < 0.01; ^∗^*p* < 0.05).

A three-way ANOVA on grammaticality accuracy, with gender of participants (female vs. male), beauty (average vs. beautiful), and gender (women vs. men) of the faces as between-participants independent variables revealed a three-way interaction, *F*(1,110) = 8.66, *p* = 0.004, $\eta_{p}^{2}$ = 0.07, H_1_ *B*_H(0,6)_ = 0.22, H_2_ *B*_H(0,6)_ = 13.33 (see **Figure 1A**). The three-way interaction was decomposed into its two-way partial interactions.

There was a beauty by gender of faces partial two-way interaction for the grammaticality accuracy of just female participants, *F*(1,58) = 4.56, *p* = 0.037, $\eta_{p}^{2}$ = 0.07, H_1_ *B*_H(0,6)_ = 0.19, H_2_ *B*_H(0,6)_ = 5.31. Female participants classified beautiful faces of women more accurately than average faces of women, with accuracy for the latter type of faces being marginally greater than chance, *t*(15) = 2.12, *p* = 0.052, *r^2^* = 0.23, *B*_H(0,6)_ = 4.16 (see **Table 2**). By contrast, female participants did not classify beautiful faces of men better than average faces of men. Thus, only female beauty enhanced women’s performance.

**Table 2:** Summary of statistics testing hypotheses about classification accuracy.

Gender of participants	Gender of faces	*F*	*p*	*df*	*$\eta_{p}^{2}$*	H_1_ *B*_H(0,6)_	H_2_ *B*_H(0,6)_
Female	Women	4.27	0.048	1, 30	0.13	4.6	0.14
	Men	1.02	0.321	1, 28	0.04	0.23	1.1
Male	Women	5.72	0.024	1, 27	0.18	0.15	8.7
	Men	0.20	0.660	1, 25	0.01	0.63	0.33

*The reported F tests represent the comparison between average and beautiful faces for each combination of gender of participants by gender of faces*.

For male participants, there was a gender of faces by beauty partial two-way interaction on grammaticality accuracy as well, *F*(1,52) = 4.10, *p* = 0.048, $\eta_{p}^{2}$ = 0.07, H_1_ *B*_H(0,6)_ = 0.23, H_2_ *B*_H(0,6)_ = 4.27 see **Figure 1A**). In contrast to female participants, who classified beautiful faces of women more accurately than average faces of women, male participants classified beautiful faces of women less accurately than average faces of women (see **Table 2**). In fact, evidence of learning was found only for average women’s faces, with males’ performance exceeding chance, *t*(12) = 1.45, *p* = 0.002, *r^2^* = 0.58, *B*_H(0,6)_ = 702.1, and not for beautiful women’s faces, where data indicated insensitivity as to whether performance exceeded chance, *t*(15) = 1.45, *p* = 0.168, *r^2^* = 0.12, *B*_H(0,6)_ = 1.49. Further, in contrast to their performance while classifying women’s faces, male participants did not classify beautiful faces of men less accurately than average faces of men. Men’s overall accuracy for male faces regardless of beauty (55%) exceeded chance, *t*(26) = 3.14, *p* = 0.004, *r^2^* = 0.28, *B*_H(0,6)_ = 40.5. Again, only female beauty impaired performance in male participants; that is, female beauty was important for both male and female participants, but affected them in opposite directions (see **Figure 1A**).

Finally, there was no opposite-sex advantage in the classification of beautiful faces for either female or male participants (both *F*s < 1 and H_1_ *B*s_H(0,6)_ < 0.33).

**Figure 1B** depicts an alternative way of illustrating the present data, with face gender being coded as same vs. opposite to participants’ gender. Analyses with the particular coding (which produces the same *F* ratios just with different effects labeled as different orders) yielded insensitive evidence of a three-way interaction (H_1_ *B*_H(0,6)_ = 0.65, H_2_ *B*_H(0,6)_ = 0.74); accordingly, no claim can be made as to whether or not the two-way interaction is different for the different genders. For a detailed interpretation of the plotting depicted in **Figure 1B**, see the Section “General Discussion.”

Overall, females demonstrated the lowest performance when classifying average faces of women, presumably because these were the least interesting faces for females. Further, female participants classified beautiful faces more accurately than average faces, even though this was true only for same-sex faces, confirming the prediction that beauty would increase perceptual learning and performance (as indicated by both *p*s and *B*s). On the other hand, male participants did not show an analogous advantage in classifying beautiful over average faces in either of the two face genders. On the contrary, and in contrast to what one would expect, males’ lowest performance was in classifying what is generally considered the most attractive type of faces for male participants, namely beautiful faces of women. Thus, male participants’ results confirmed the prediction that high incentive salience would interfere with perceptual processing (again confirmed by both *p*s and *B*s). Overall, female beauty influenced classification accuracy in both males and females, but in exactly the opposite direction, and in a way that male beauty did not.

Finally, the overall pattern of classification performance suggests that AGL performance involves more than a pure liking process, that is, more than what is involved in an esthetic judgment task, as there is ample evidence that men and women agree on attractiveness judgments (e.g., Langlois et al., 2000; Kranz and Ishai, 2006; Ishai, 2007; Levy et al., 2008).

### Subjective Measures of Awareness

The analysis of participants’ accuracy for all the attributional categories was used to measure the conscious status of participants’ knowledge. The knowledge of the structure of the training items that participants acquire constitutes the *structural knowledge* and may consist of different knowledge types (e.g., knowledge of fragments or of whole items, knowledge of other rules, etc.). Here we make no specific assumptions as to the exact content of participants’ structural knowledge. The knowledge of whether test items have the structure of training items forms participants’ *judgment knowledge* (see Dienes and Scott, 2005 about the distinction between structural and judgment knowledge). The analyses of participants’ knowledge attributions allow us to make conclusions about the consciousness of both structural and judgment knowledge. Guessing attributions correspond to unconscious structural and unconscious judgment knowledge. Familiarity and intuition attributions reflect unconscious structural and conscious judgment knowledge. Finally, rules and memory attributions reflect conscious structural and conscious judgment knowledge. The above three possible categories of the (un)consciousness of knowledge will be followed while analyzing participants’ knowledge attributions. First, we present the percentage of all attribution categories.

#### Frequency of Knowledge Attributions

**FIGURE 2:**
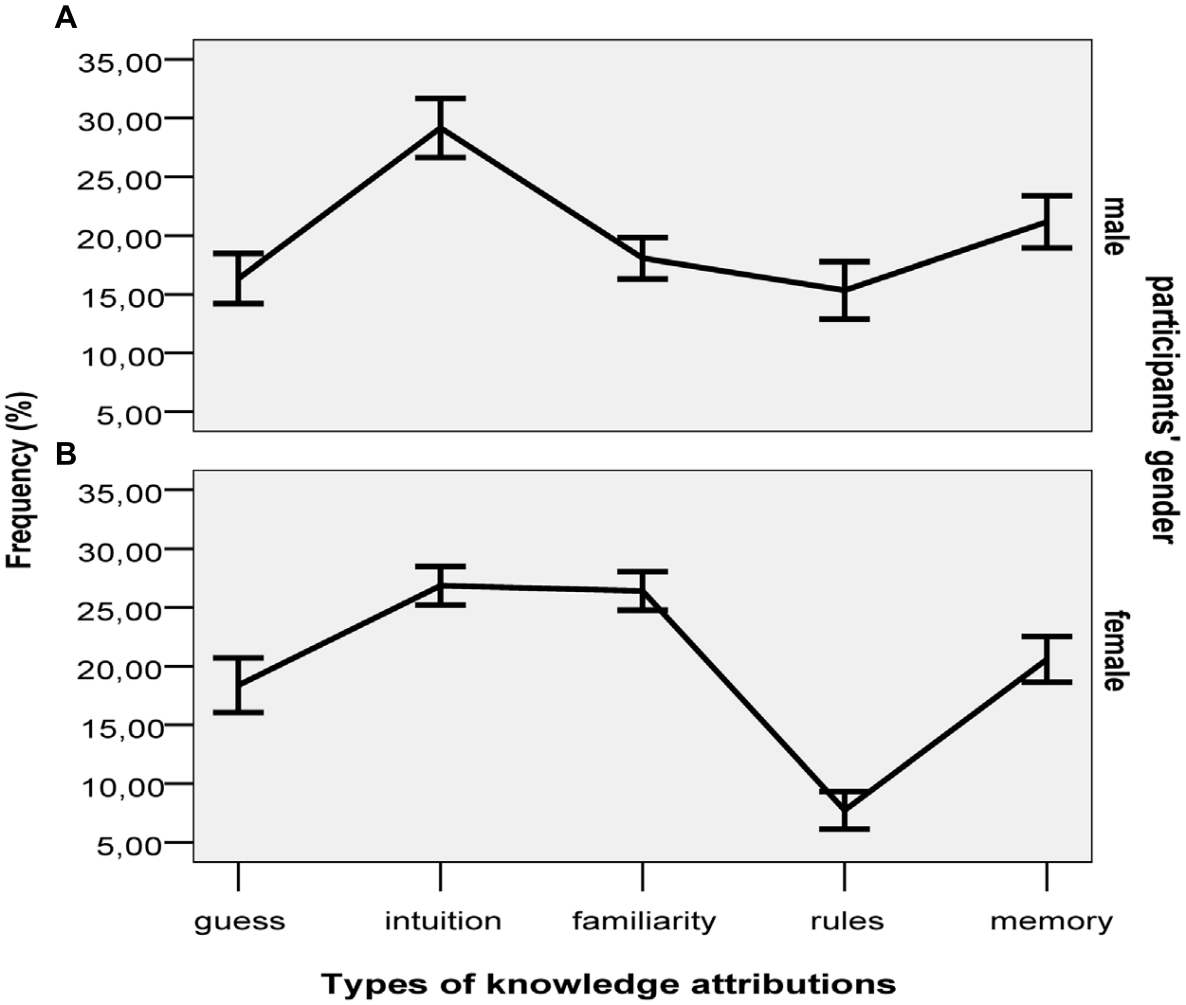
**Percentage of knowledge attributions in males (A) and females (B).** Error bars indicate ±1 SE.

The guess, intuition and familiarity knowledge attributions were combined to create implicit attribution scores, and rules and memory attributions were combined to create explicit attribution scores. A three-way ANOVA on percentage of implicit attributions, with gender of participants, face gender, and beauty (average vs. beautiful) as independent variables revealed only a significant main effect of participants’ gender, *F*(1,110) = 5.44, *p* = 0.022, $\eta_{p}^{2}$ = 0.05, *B*_N(0,15)_ = 3.19. Female participants gave significantly more implicit attributions than male participants (72% vs. 63%). The frequencies of all five knowledge attributions for the two genders are shown in **Figure 2**.

(For the estimation of *B* in this case, the alternative was modeled as a normal centered on zero with an SD of 15, i.e., the predictions were not directional. Setting the SD to this value implies that changes up to 30% in either direction are plausible; and that is about as large as the changes could be, keeping predictions symmetrical, given mean values of around 70%.)

#### The (Un)Consciousness of Knowledge

Only 62 of the 118 participants had classification accuracy data in all five attributions. Therefore, the three implicit attributions (guess, intuition, and familiarity) were collapsed, and the same was done for the two explicit ones (rules and memory), which resulted in an ANOVA with *N* = 110 participants. The four-way ANOVA on accuracy with gender of participants, gender of faces, beauty (average vs. beautiful), and attribution type (implicit vs. explicit) as independent variables showed only a significant effect of attribution, *F*(1,102) = 18.58, *p* < 0.001, $\eta_{p}^{2}$ = 0.15, *B*_H(0,10)_ = 2854.80. (Dienes and Scott, 2005, found an effect of attribution of 10%; this value was used for modeling the predictions of the alternative hypothesis while calculating *B* in this case.) Explicit attributions were accompanied by significantly higher accuracy than implicit attributions (61% vs. 54%). Accuracy was significantly higher than chance in both the former, *t*(109) = 8.02, *p* < 0.001, *r^2^* = 0.37, *B*_H(0,6)_ = 9.0^∗^10^12^, and the latter attribution types, *t*(109) = 4.83, *p* < 0.001, *r^2^* = 0.18, *B*_H(0,6)_ = 24349.00. Thus, participants acquired both implicit and explicit structural knowledge.

In order to get a clearer picture of participants’ implicit and explicit knowledge, we analyzed accuracy for the different knowledge attributions relying on the different combinations of unconscious and conscious structural and judgment knowledge as these were defined above.

##### Unconscious structural and unconscious judgment knowledge

**FIGURE 3:**
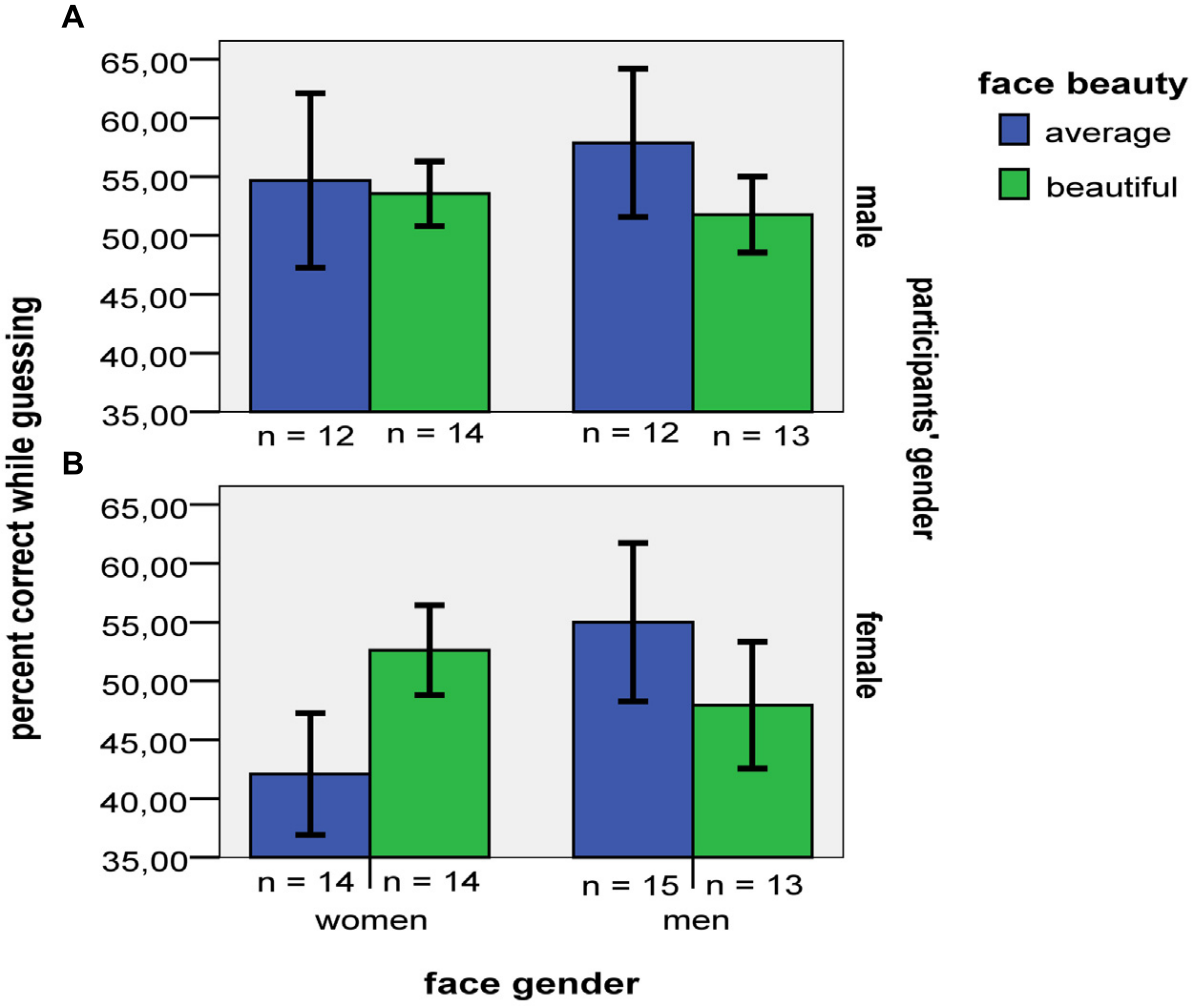
**Mean accuracy when males **(A)** and females **(B)** provided unconscious structural and judgment knowledge (i.e., guess) attributions in the four different stimulus categories.** Error bars indicate ±1 SE.

The 2 × 2 × 2 [gender of participants (males vs. females) by gender of faces (women vs. men) by beauty (average vs. beautiful)] analysis of accuracy just for guess attributions revealed no significant effects or interactions, *F*s < 2, *p*s > 0.10 (see **Figure 3**).

The data were insensitive for indicating whether or not participants’ overall accuracy for guess attributions (52%) exceeded chance, *t*(106) = 0.99, *p* = 0.324, *r^2^* = 0.01, *B*_H(0,6)_ = 0.77.

##### Unconscious structural and conscious judgment knowledge

As previously stated, H_1_ *B* tests the hypotheses that performance will be higher for beautiful than for average faces and H_2_ *B* the opposite. For two-way interactions, H_1_ and H_2_ test whether learning is greatest for beautiful faces of different than of same gender and of same than of different gender respectively. For the three-way interaction, H_1_ tests whether males demonstrate an advantage for beautiful over average faces of women rather than men as compared to female participants. H_2_ represents the opposite direction.

**FIGURE 4:**
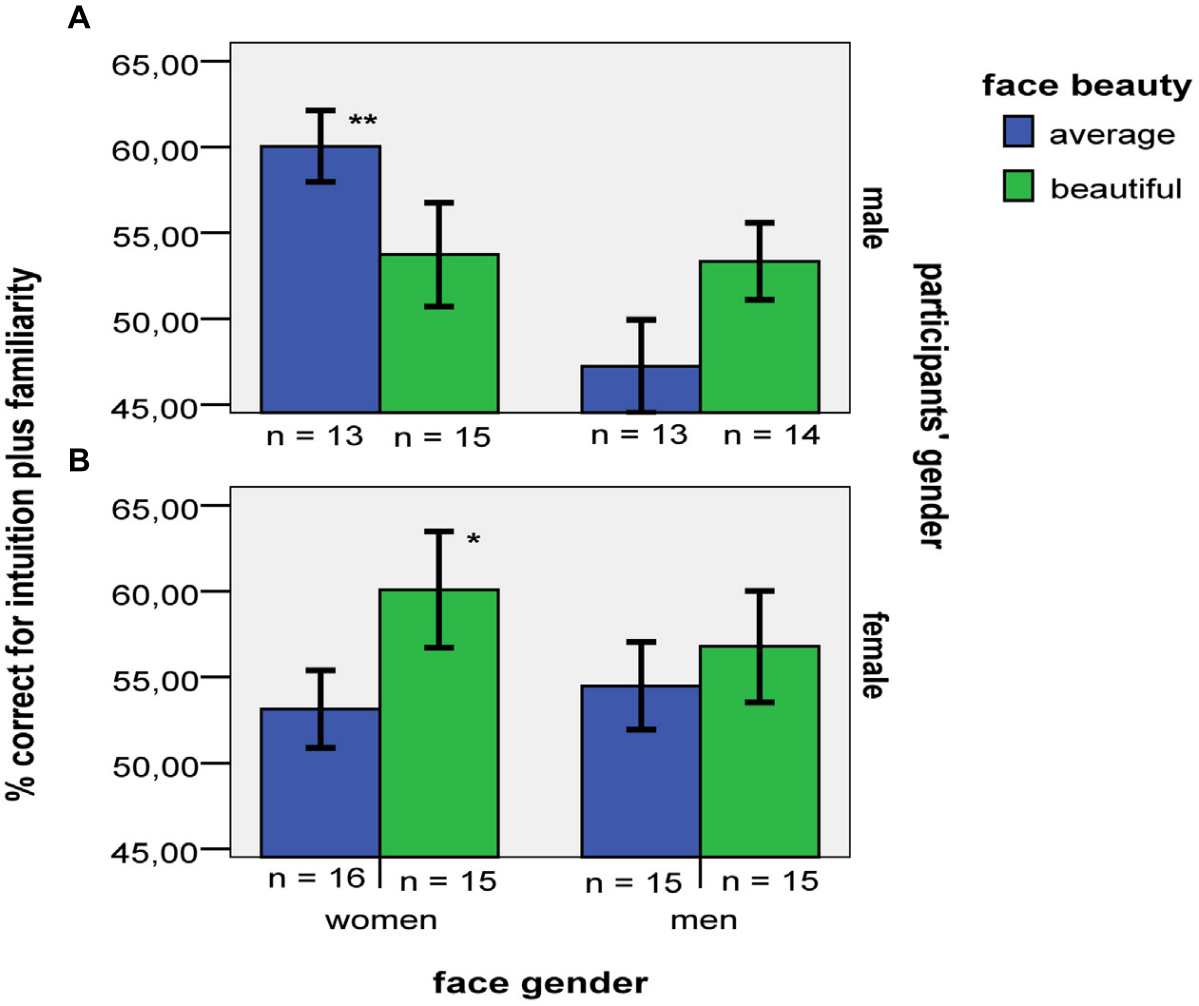
**Mean accuracy when males **(A)** and females **(B)** provided unconscious structural knowledge (i.e., intuition and familiarity) attributions in the four different stimulus categories.** Error bars indicate ±1 SE. Asterisks denote significant differences from a chance level of 50% (^∗∗^*p* < 0.01; ^∗^*p* < 0.05).

The three-way analysis of accuracy for only intuition and familiarity attributions combined together revealed a three-way interaction, *F*(1,108) = 4.79, *p* = 0.031, $\eta_{p}^{2}$ = 0.04, H_1_ *B*_H(0,6)_ = 0.35, H_2_ *B*_H(0,6)_ = 3.52 (see **Figure 4**). The three-way interaction was decomposed into partial two-way interactions. The analysis of female participants’ data showed insensitive evidence for a gender of face by beauty two-way interaction, *F*(1,57) = 0.66, *p* = 0.42, $\eta_{p}^{2}$ = 0.01, H_1_ *B*_H(0,6)_ = 0.46, H_2_ *B*_H(0,6)_ = 1.19. Their overall accuracy for intuition and familiarity combined together (56%) exceeded chance, *t*(60) = 4.21, *r^2^* = 0.23, *p* < 0.001, *B*_H(0,6)_ = 2754, providing evidence of unconscious structural knowledge.

The analysis of male participants’ accuracy for only intuition and familiarity attributions combined together yielded a gender of faces by beauty interaction, *F*(1,51) = 5.77, *p* = 0.020, $\eta_{p}^{2}$ = 0.10, H_1_ *B*_H(0,6)_ = 0.24, H_2_ *B*_H(0,6)_ = 6.39 (see **Figure 4**). Male participants were not more accurate in their implicit knowledge for beautiful rather than average faces of women, (see **Table 3**). Further, their implicit knowledge was not less accurate for beautiful than average faces of men. Finally, males’ data were insensitive for determining an effect of face gender for beautiful faces, *F*(1,27) = 0.01, *p* = 0.917, $\eta_{p}^{2}$ < 0.001, H_1_ *B*_H(0,6)_ = 0.58, H_2_ *B*_H(0,6)_ = 0.50, or whether males’ implicit knowledge accuracy for beautiful faces overall (54%) exceeded chance, *t*(28) = 1.90, *p* = 0.068, *r^2^* = 0.11, *B*_H(0,6)_ = 2.7.

**Table 3:** Summary of statistics testing hypotheses about unconscious and conscious structural knowledge.

Gender of participants	Gender of faces	*F*	*p*	*df*	$\eta_{p}^{2}$	H_1_ *B*_H(0,6)_	H_2_ *B*_H(0,6)_
**Unconscious structural knowledge**					
Female	Women	–	–	–	–	–	–
	Men	–	–	–	–	–	–
Male	Women	2.77	0.108	1, 26	0.10	0.23	2.65
	Men	3.05	0.093	1, 25	0.11	2.93	0.21
**Conscious structural knowledge**					
Female	Women	4.06	0.054	1, 27	0.13	4.04	0.25
	Men	3.86	0.060	1, 26	0.13	0.34	3.13
Male	Women	–	–	–	–	–	–
	Men	–	–	–	–	–	–

*Unconscious structural knowledge here includes only the combination of intuition and familiarity attributions. Blank cells indicate insensitive evidence of two-way interactions, which were thus not followed by one-way ANOVAs*.

In sum, both males and females acquired unconscious structural knowledge. Importantly, for male participants, beauty impaired the unconscious structural knowledge of women’s faces more than men’s faces. Further, the extent to which this happened for males was greater than for females (for whom it may even have gone in the other direction). Thus, beauty affected the unconscious structural knowledge of males and females differently.

##### Conscious structural and conscious judgment knowledge

Again, note that H_1_ tests whether there is an advantage for beautiful over average faces and H_2_ the opposite. For two-way interactions, H_1_ and H_2_ test whether learning is greatest for beautiful faces of different than of same gender and of same than of different gender respectively. For the three-way interaction, H_1_ tests whether males demonstrate an advantage for beautiful over average faces of women rather than men as compared to females. H_2_ represents the opposite direction.

**FIGURE 5:**
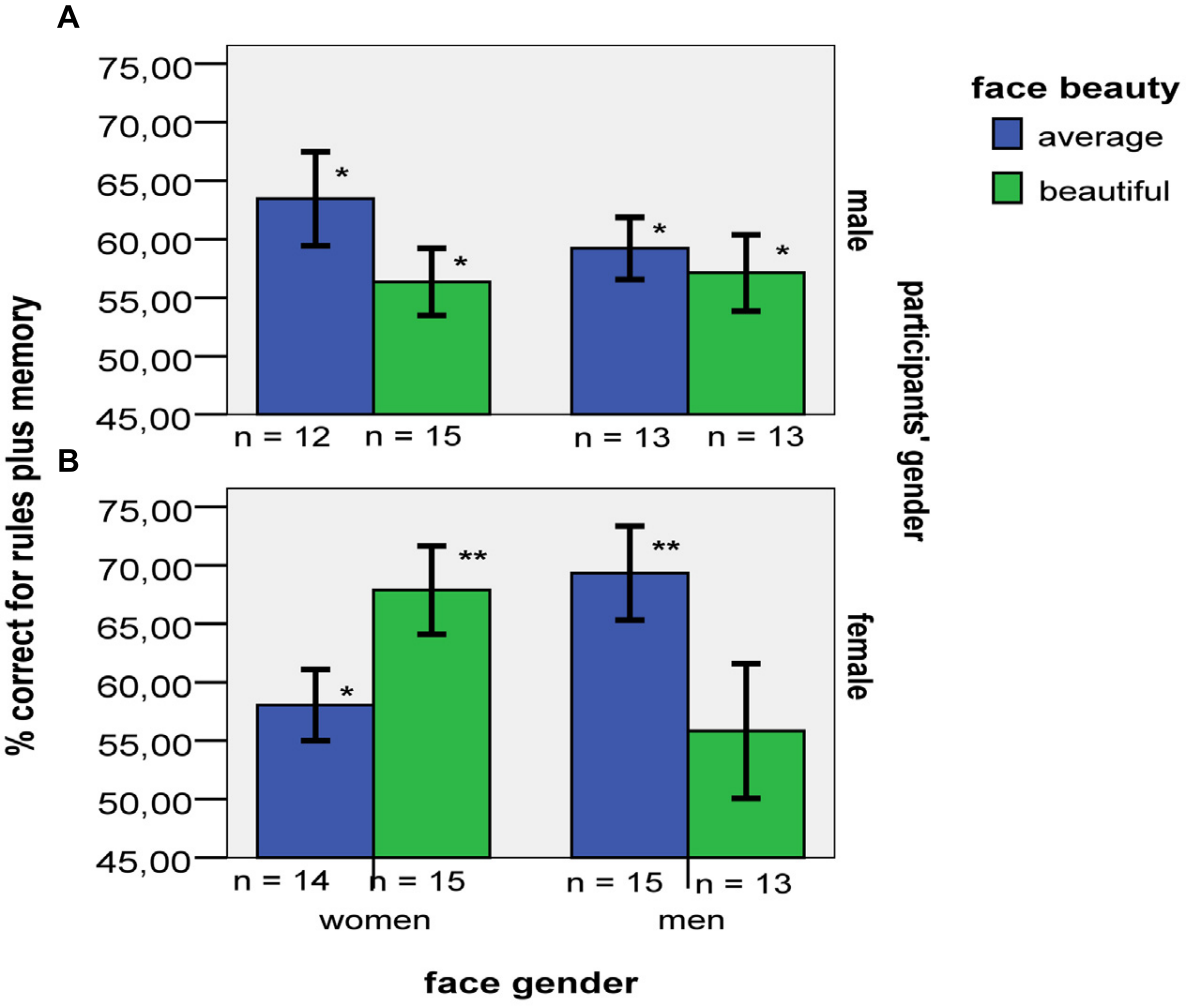
**Mean accuracy when males **(A)** and females **(B)** provided conscious structural knowledge (i.e., rules and memory) attributions in the four different stimulus categories.** Error bars indicate ±1 SE. Asterisks denote significant differences from a chance level of 50% (^∗∗^*p* < 0.01; ^∗^*p* < 0.05).

The analysis of accuracy just for rules and memory attributions combined together revealed a significant three-way interaction, *F*(1,108) = 7.05, *p* = 0.009, $\eta_{p}^{2}$ = 0.10, H_1_ *B*_H(0,6)_ = 0.40, H_2_ *B*_H(0,6)_ = 3.64 (see **Figure 5**).

As before, the three-way interaction was decomposed into partial two-way interactions. The analysis of female participants’ data yielded a face gender by beauty interaction on accuracy of explicit structural knowledge, *F*(1,53) = 7.75, *p* = 0.007, $\eta_{p}^{2}$ = 0.13, H_1_ *B*_H(0,6)_ = 0.32, H_2_ *B*_H(0,6)_ = 5.68. Females’ accuracy of explicit knowledge for beautiful faces of women was higher than the corresponding accuracy for average faces of women (marginal by significance test; see **Table 3**). Further, females’ accuracy of explicit knowledge was (marginally) lower for beautiful than average faces of men. There was no substantial evidence of a face gender effect on females’ explicit knowledge of beautiful faces, *F*(1,26) = 3.22, *p* = 0.084, $\eta_{p}^{2}$ = 0.11, H_1_ *B*_H(0,6)_ = 0.35, H_2_ *B*_H(0,6)_ = 2.72.

The analysis of male participants’ accuracy of explicit structural knowledge yielded insensitive evidence for an interaction, *F*(1,49) = 0.61, *p* = 0.439, $\eta_{p}^{2}$ = 0.01, H_1_ *B*_H(0,6)_ = 0.50, H_2_ *B*_H(0,6)_ = 1.18. Their overall accuracy for rules and memory attributions combined together (59%) exceeded chance, *t*(52) = 5.54, *p* < 0.001, *r^2^* = 0.37, *B*_H(0,6)_ = 965736.

Overall, both males and females acquired conscious structural knowledge of faces. Further, in line with their classification accuracy, females’ explicit structural knowledge for beautiful faces of women was higher than the corresponding accuracy for average faces of women. For women, the facilitatory effect of beauty on conscious structural knowledge for female faces was greater than for male faces. Further, the extent of this effect was greater for female than male participants (who may even have gone in the opposite direction). Beauty affected the conscious structural knowledge of males and females differently.

## General Discussion

The present work aimed at exploring the effect of facial attractiveness, that is, of stimuli with high incentive salience on the acquisition of implicit and explicit knowledge acquired in an AGL task. Our findings are discussed with respect to theoretical views about possible gender differences in the way and the degree in which same- and opposite-sex facial attractiveness captures people’s attention and thus determines the incentive salience of attractiveness. Accordingly, the discussion also touches upon the relation of attention and AGL, without of course seeking or being able to provide conclusive answers about this intricate relation.

The overall pattern of findings revealed that (opposite-sex) facial attractiveness interfered with male participants’ classification accuracy and unconscious knowledge. In particular, the classification performance results showed that male participants demonstrated the lowest accuracy while classifying beautiful faces of women. Further, beauty impaired the unconscious structural knowledge (i.e., accuracy in the combination of intuition and familiarity attributions) of women’s faces more than men’s faces for male participants. This occurred to a greater extent for males than for females. Conversely, female participants demonstrated greater classification accuracy and explicit knowledge for beautiful than for average faces of the same sex. First of all, opposite-sex attractiveness as well as same-sex attractiveness may exert different motivating influence on men and women and consequently lead to different cognitive processing modes in the two genders, a possibility that is examined in detail below. Secondly, a related issue concerns the effect that attention generally demonstrated to be captured by attractive faces (e.g., Langlois et al., 1987, 1991; Maner et al., 2003) has on perceptual processing and more specifically on implicit and explicit AGL. The two issues are examined in turn below.

### The Effect of Motivational Gender Differences on (Un)Consciousness

Maner et al. (2003; see also Neuberg et al., 2004) have summed up previous research on mating-relevant goals in the two genders under the following evolutionary-based hypotheses concerning gender differences in selective processing of attractive men and women. The *opposite-sex beauty captures the eye* hypothesis is in line with theoretical and empirical evidence than both genders exhibit great sensitivity toward opposite-sex attractiveness. According to the *one-sided gender bias* hypothesis, men are more likely to selectively focus on opposite-sex attractiveness than are women. Finally, on the *female beauty captures the eye* hypothesis, both genders selectively attend to attractive faces of women for different reasons: men motivated by mate-search motives and women because they view women as competitors.

The current research suggests that incentive salience instantiated by attractive faces of same and opposite sex exerts different influence on cognitive processing. With respect to opposite-sex attractiveness, in particular, the present findings are consistent with hypotheses and corresponding empirical evidence suggesting that men and women value physical attractiveness in a different way when selecting mates. Several studies have shown that men exhibit greater sensitivity to viewing attractive faces of the opposite sex than women do (Hayden et al., 2007). Further, it has been found that males, but not females, are willing to discount higher future rewards for smaller immediate ones when they view attractive faces of the opposite sex (Wilson and Daly, 2004). In addition, Levy et al. (2008) have shown that men exerted greater motivational effort for viewing beautiful images of the opposite sex than did women. Along the same lines, it has been suggested that, when selecting mates, men rely on attractiveness whereas women give emphasis on resources (e.g., Sadalla et al., 1987; Buss, 1989, 1999; Li et al., 2002; see also Cloutier et al., 2008 for evidence of fMRI activation of OFC, i.e., a reward-related brain region, during processing facial attractiveness, only in male participants). On this mate selection theory, the present findings suggest that the increased motivational value that opposite-sex beauty has for men leads to a detrimental effect on their performance in an AGL task. In particular, there was evidence of opposite-sex beauty impairing mostly male participants’ performance. In particular, opposite-sex beauty interfered with male participants’ classification accuracy. Further, beauty impaired their unconscious structural knowledge of women’s faces more than men’s faces. The above difference was greater for male than for female participants (for whom the above difference may even have gone in the other direction).

Another possible difference between the two genders concerns the effect of same-sex attractiveness on performance. Evidence of an effect of same-sex beauty was found only in females’ and not in males’ performance. In particular, female participants demonstrated higher classification accuracy and a (marginally) greater amount of explicit structural knowledge for beautiful over average faces of women.

However, the above differences with respect to the effect of same- and opposite-sex attractiveness on the performance of males and females, at the same time, point to a similar pattern in the performance of the two genders in that the data of both males and females provide converging evidence of the *female beauty captures the eye* hypothesis. Indeed, both genders demonstrated the sensitivity toward female beauty that the above hypothesis entails. In particular, female participants demonstrated greater classification accuracy and explicit knowledge for beautiful than for average faces of women. On the other hand, males demonstrated a reduced classification accuracy for beautiful over average faces of women. Further, beauty impaired their unconscious structural knowledge of women’s faces more than men’s faces. Thus, it seems that female beauty captured the attention of both genders, with the difference that in females it led to an advantage, whereas in men to a disadvantage. It has been suggested that not only men but also women demonstrate an increased selective attention and recognition memory for attractive faces of women (e.g., Shepherd and Ellis, 1973; Maner et al., 2003; Becker et al., 2005). These findings are consistent with females’ increased explicit knowledge of attractive faces of women (as measured by accuracy for rules and memory attributions). The salience of attractive women for other women may originate from a mate value self-assessment motive or from a motive of mate guarding from potential competitors (cf. Buss and Shackelford, 1997; Gutierres et al., 1999). The latter possibility is consistent with Becerra et al. (2001) findings suggesting that there may be a shared neural system for the evaluation of aversive and rewarding stimuli. By contrast, the salience of attractive women for men may originate from a mate-search motive.

Apart from the *female beauty captures the eye* hypothesis, the present findings are consistent with the *one-sided gender bias* hypothesis, according to which men are more likely to selectively focus on opposite-sex beauty than are women. By contrast, our findings do not support the *opposite-sex beauty captures the eye* hypothesis, according to which both genders should demonstrate great sensitivity toward opposite-sex attractiveness.

The difference in the way same-sex attractiveness as well as opposite-sex attractiveness affected the performance of the two genders may be explained by research providing evidence that women do not process opposite- and same-sex stimuli in the manner men do (e.g., Maner et al., 2003; Cloutier et al., 2008; Franklin and Adams, 2009; see also Rupp and Wallen, 2008). Thus, attractive faces might have a different reward value for men and women. Alternatively, it could be argued that women rely more on esthetic aspects of attractiveness of both sexes (which may or may not have a sexual reward value cf. Franklin and Adams, 2009), whereas men focus more on sexual reward-based aspects of stimuli. The above dissociation resembles the dissociation between “liking” and “wanting” described by Berridge (2000) and might be responsible for the gender differences in the processing of attractive men and women.

An alternative interpretation of the present data is also plausible. We found that for men opposite-sex beautiful vs. average faces impaired performance. For women, the same contrast was insensitive. Thus, it remains open whether or not for women opposite-sex beautiful vs. average faces impaired performance. According to the *female beauty captures the eye* hypothesis, the null hypothesis is true for this last contrast. But the evidence allows the null to be true or false. Similarly, we found that for women same-sex beautiful vs. average faces facilitated performance. For men, the same contrast was insensitive. Thus, it remains open whether or not for men same-sex beautiful vs. average faces facilitated performance. According to the *female beauty captures the eye* hypothesis, the null hypothesis is true for this last contrast. But the evidence allows the null to be true or false. **Figure 1B** emphasizes the extent to which for opposite-sex faces, beauty seems to inhibit performance, whereas for same-sex ones the opposite is true. The above differences may be independent of participants’ gender. Overall, it could be argued that the increased incentive salience of opposite-sex attractiveness functions as a distractor that eliminates perceptual resources, thereby harming performance. By contrast, attractive faces of the same sex (as well as average faces of the same sex) presumably have a lower incentive salience that broadens attentional and cognitive resources, thereby enhancing learning.

In sum, the theory that relates high and low motivational intensity with the narrowing or broadening of attentional and cognitive resources respectively (Gable and Harmon-Jones, 2010b) may apply to both genders equally. Further research is needed to distinguish this theory from the *female beauty captures the eye* theory, as both survive the tests in this study.

An alternative mechanism that might have played a specific or greater role in the influences of opposite-sex attractiveness on AGL performance is based on the effect of beauty on implicit preferences. A well-established finding in the implicit learning literature is an increased liking for grammatical over ungrammatical strings of an AG (see e.g., Zizak and Reber, 2004). Such implicit preferences could have facilitated classification performance in the test phase. However, test strings that consist of attractive faces of the opposite sex, namely of faces that are thought to be generally preferred might have interfered with participants’ implicit preferences guiding their judgments, which in turn could account for a disadvantage in the classification of attractive over average faces of the opposite sex.

### Attractiveness, Attention, and Cognitive Processing

The predictions of the present study were formulated on the basis of the possible ways in which the incentive salience of attractive faces might guide attention, which in turn could play a central role in the acquisition of implicit and explicit knowledge. On the one hand, it was hypothesized that if incentive salience increased perceptual processing, implicit learning would increase for attractive rather than average faces, especially of the opposite sex. On the other hand, if incentive salience narrowed the breadth of perceptual processing, with participants selectively attending to arousing information, like the physical characteristics of beautiful faces rather than to the task-relevant order of faces, performance might be impaired.

Male participants’ reduced classification accuracy for attractive faces of women as well as the greater interference effect that beauty had on unconscious structural knowledge of women’s faces rather than men’s faces for males as compared to females supports the second hypothesis and theoretical and empirical data that are consistent with it. Thus, according to Easterbrook’s (1959) hypothesis, arousing stimuli reduce the range of perceptual focus (by excluding less arousing, irrelevant, or peripheral information). As Easterbrook (1959) notes, this narrowing of attention caused by emotional arousal can have a positive effect in some tasks, but, in most cases, it inhibits performance. It should be noted that Easterbrook’s (1959) hypothesis related to negative affect only. However, other researchers (Gable and Harmon-Jones, 2008, 2010a,b; Harmon-Jones and Gable, 2009; see also Levine and Edelstein, 2009) have shown that stimuli with high motivational intensity reduce the breadth of attention, in both withdrawal motivation (inherent to negative affect stimuli) and in approach motivation (inherent to positive affect stimuli). Further, it has been shown that men are more likely to drift into sexual fantasies by an external trigger (e.g., relevant visual stimuli) than women are (Ellis and Symons, 1990; Jones and Barlow, 1990). Accordingly, male participants might have had a reduction in the visual resources/perceptual attention toward attractive female faces, which might explain their low classification (and implicit knowledge) accuracy for these faces.

This attentional narrowing during the processing of emotional and highly motivating stimuli (attractive faces), which presumably interfered with perceptual processing and accordingly with implicit learning, could at the same time be associated with an elaborate processing of the stimuli or dimensions attended to, which, in turn, might explain male participants’ intact explicit knowledge for these faces. Consistently, many studies have found enhanced memory for emotional stimuli (see Levine and Edelstein, 2009, for a review of such studies).

Along similar lines to the attentional narrowing hypothesis, is a theoretical interpretation derived from the research field of AGL, which also gives emphasis on the exclusion of goal irrelevant information during learning. In particular, Tanaka et al. (2008), Eitam et al. (2009) and Kiyokawa et al. (2012) provide strong evidence that directing participants’ attention to goal relevant dimensions of the stimuli is a prerequisite for implicit AGL. Instructing participants to attend to stimuli may be one way of rendering stimuli goal relevant (Eitam et al., 2009), but it is not the only one. Reward-based sexual processes may be viewed as another way of relating stimuli to people’s goals. Thus, men’s selective attention might have focused on goal- and reward-relevant physical characteristics of beautiful faces (i.e., on irrelevant dimensions of the task), which might have interfered with attention on the relevant aspects of the task (e.g., the order of faces), and accordingly with AGL in general and with implicit learning in particular.

Female participants’ performance, and in particular, their processing advantage for attractive over average faces of women (in terms of their classification accuracy and their explicit structural knowledge) is consistent with the hypothesis that incentive salience (in this case originating from a mate-guarding motive) would increase perceptual processing and accordingly performance in the AGL task. If females focused more on the esthetic rather than the motivational aspect of beauty while attending to attractive faces of women, this might have led to the broadening of attention. Indeed, it has been shown that it is the motivational aspect (i.e., desire) of positive affect that leads to attentional narrowing, whereas positive affect of low approach motivation (e.g., pleasure) leads to attentional and cognitive broadening (Gable and Harmon-Jones, 2008).

The increase in female participants’ explicit knowledge while classifying attractive faces of females and their intact implicit knowledge is also consistent with the hypothesis that incentive salience would increase executive processing, that is attentional resources rather than perceptual resources. Further, as mentioned above, this increased explicit knowledge is consistent with research showing that women (like men) demonstrate an increased selective attention and recognition memory for attractive female faces (e.g., Shepherd and Ellis, 1973; Maner et al., 2003).

Finally, the fact that female participants demonstrated the opposite pattern for explicit knowledge of men’s faces (with marginally decreased explicit knowledge for attractive relative to average male faces) may be due to a difference in women’s strategies while processing facial attractiveness of the two sexes, and in particular, in the balance between the esthetic and sexual reward value involved in this processing.

It should be noted again that all the above interpretations concerning the effect of beauty on the implicit and explicit processing of same- and opposite-sex faces in each participants’ gender might hold true for both genders. The present data cannot rule out either of the two possibilities.

## Conclusion

This study examined whether the processing of incentive salience instantiated by facial attractiveness leads to explicit and/or implicit knowledge acquired in an AGL task and whether this processing is differentially affected by the gender of participants depending on the sex of the attractive faces. Our findings revealed both gender similarities and differences in the processing of facial attractiveness. More specifically, the overall pattern of participants’ performance suggests that female beauty captured the attention of both genders. However, this increased sensitivity toward female beauty affected performance of the two genders in opposite directions. On the one hand, the overall performance of male participants suggests that incentive salience interferes with perceptual processing, presumably because the increased rewarding value that attractive faces of women has for men narrows the breadth of perceptual focus. Such an attentional narrowing interfered with performance in AGL and in particular with implicit learning, which has been shown to rely on perceptual resources (e.g., Tanaka et al., 2008; Eitam et al., 2009). On the other hand, the increased classification accuracy and explicit structural knowledge that female participants demonstrated for attractive over average faces of women, that is for motivationally salient stimuli depicting potential competitors for women is in line with the hypothesis that incentive salience would increase perceptual processing as well as attentional resources. Presumably, the two genders differ in the degree to which they rely on esthetic and rewarding aspects of beauty or on liking and wanting processes, which in turn might differentially affect conscious and unconscious processing. Future work could identify the mechanisms allowing same- or opposite-sex attractiveness to exert such a different influence on the acquisition of conscious and unconscious knowledge in men and women, thereby illuminating the intriguing relation of consciousness and motivating stimuli with important cognitive and societal implications.

## Conflict of Interest Statement

The authors declare that the research was conducted in the absence of any commercial or financial relationships that could be construed as a potential conflict of interest.
